# Factors Affecting Species Identifications of Blow Fly Pupae Based upon Chemical Profiles and Multivariate Statistics

**DOI:** 10.3390/insects8020043

**Published:** 2017-04-11

**Authors:** William Kranz, Clinton Carroll, Darren A. Dixon, John V. Goodpaster, Christine J. Picard

**Affiliations:** 1 Department of Chemistry and Chemical Biology, Indiana University Purdue University Indianapolis, Indianapolis, IN 46202, USA; wkranz@umail.iu.edu (W.K.); clincarr@iupui.edu (C.C.); darrdixo@iupui.edu (D.A.D.); 2 Forensic and Investigative Sciences Program, Indiana University Purdue University Indianapolis, Indianapolis, IN 46202, USA; 3 Department of Biology, Indiana University Purdue University Indianapolis, Indianapolis, IN 46202, USA

**Keywords:** *Phormia regina*, *Lucilia sericata*, *Lucilia cuprina*, *Cochliomyia macellaria*, forensic entomology, TV-SPME, blow fly pupae, lipid analysis

## Abstract

Alternative methods for the identification of species of blow fly pupae have been developed over the years that consist of the analyses of chemical profiles. However, the effect of biotic and abiotic factors that could influence the predictive manner for the tests have not been evaluated. The lipids of blowfly pupae (*Cochliomyia macellaria*, *Lucilia cuprina*, *Lucilia sericata*, and *Phormia regina*) were extracted in pentane, derivatized, and analyzed by total-vaporization solid phase microextraction gas chromatography-mass spectrometry (TV-SPME GC-MS). Peak areas for 26 compounds were analyzed. Here we evaluated one biotic factor (colonization) on four species of blow flies to determine how well a model produced from lipid profiles of colonized flies predicted the species of flies of offspring of wild-caught flies and found very good species identification following 10 generations of inbreeding. When we evaluated four abiotic factors in our fly rearing protocols (temperature, humidity, pupation substrate, and diet), we found that the ability to assign the chemical profile to the correct species was greatly reduced.

## 1. Introduction

Insects make up a large proportion of the environment; therefore, it is not surprising that insects would be present in crime scenes. Insects play an important role in decomposition, therefore they become forensically useful in estimating the postmortem interval [1]. Insects recovered from the body can be used as a “clock” to determine the minimum postmortem interval (PMI_MIN_), or the time since the body has been available to the insects, as most species in North America will only lay eggs or larvae on a resource if it is dead [2,3].

The most common fly that will be encountered in casework is the blowfly (Diptera: Calliphoridae). The species of the collected specimens is determined, and some measure of their age—usually the length of the larvae or the life cycle stage of the insects’ development—is used to extrapolate PMI_MIN_, referencing published developmental data (e.g., [4,5,6,7,8,9]). To estimate the PMI_MIN_, investigators should attempt to collect the oldest insect specimens present at the crime scene, and in some cases, these are pupae. Due to their lack of morphological characters, it is often difficult, if not impossible, to identify the species. Therefore, pupae are typically placed in an environmental chamber at known temperature and allowed to complete their development. Once they are adults, the species can be determined using published morphological keys [10]. Two shortcomings of this technique are the additional time needed for the pupae to complete development, as well as the assumption that the pupae are still viable. Any damaged or dead pupae will never be identified by this method, and few crime laboratories have the resources available to rear flies. An alternative is to extract the DNA from the pupae and determine the species typically by sequencing a mitochondrial DNA locus [11,12,13,14,15,16,17], however, expertise is necessary for the analysis of the DNA sequence, and a thorough representation of all possible species is required [18].

Further complicating the use of blow fly pupae for forensic postmortem interval estimations is the process of estimating the age of the pupae. Various methods have been proposed, which include an analysis of the differential expression of genes [19,20]. However, this requires a well-preserved, high-quality sample with intact mRNA. Other proposed methods include the analysis of the internal morphology of the pupa [21,22,23], however, this requires a great deal of expertise, and has only been done on a single species to date.

Considering these issues, experts in the field of forensic entomology have suggested chemical analyses to complement, or substitute for, traditional morphological examinations [2] and/or DNA-based methods. Such chemical analyses would be easily conducted on instruments common to all forensic laboratories, alleviating the burden on smaller labs. They would also be more objective, subject to empirical scrutiny, and able to report on their own propensity for error.

Several possible methods of analysis have been proposed in recent years, but the idea that has shown the most promise relies upon the analysis of the organic compounds that can be extracted from the insect cuticle [2]. Associated with the cuticle are a multitude of chemicals—hydrocarbons, free fatty acids, alcohols, aldehydes, wax esters, and fatty acid methyl esters [24]—which serve a variety of purposes with respect to the insect’s physiology and life cycle.

All insects have cuticular hydrocarbons, those hydrocarbons can often be individualized to species—or, even further, to the sex of a species, to a part of the life cycle, or to a caste [25]. The evolution of such compounds, which include long-chain hydrocarbons and hydrophobic moieties, appears to have originated from the need, by insects, to retain water within their bodies and prevent desiccation [26]. This knowledge is supported by the observation that species of Drosophila found in arid environments have a complement of hydrocarbons whose chains are longer than those in more temperate climates [27,28]. In addition to this water retention function, the cuticular compounds serve to protect the insect in several other ways—notably, by providing a barrier to infiltration by insecticides and toxins [29], as well as fungi and bacteria [24]. The hydrocarbons of the cuticle can also convey information via chemical signaling. When one insect encounters another, the cuticular hydrocarbon profile may signal any or all of the following: (i) that the individual is a member of the same species [25]; (ii) of the same colony; (iii) male or female [30]; (iv) caste membership [31]; (v) whether the individual is a close family relation, or a dominant member of the colony attempting to pass along an order, or an inferior member standing by to take a command [32]; and (vi) whether the other insect is fertile and ready to mate, or has already copulated [33]. Flies are able to detect cuticular hydrocarbons via the olfactory organs of the maxillary palps and antennae, or by taste, via the organs of the proboscis [28].

Likewise, a constellation of fatty acids and sterols have been isolated from flies, as described by Golebiowski et al. [24,33,34]. Free fatty acids ranging between hexanoic acid (6:0) and hexacosanoic acid (26:0) have been described, although many of these are at trace levels in comparison to the dominant palmitic acid, palmitoleic acid, oleic acid, linoleic acid, and stearic acid contributions . Like the hydrocarbons, these lipids are known to provide resistance to desiccation, fungal infection, and bacterial infection. A number of sterols have also been reported, including cholesterol, campesterol, and sitosterol, although cholesterol appears to be the most consistently dominant [35].

As there appear to be qualitative and quantitative differences in the occurrence of these compounds among the various species, the chemical profiles may be useful as a tool for identification and discrimination. Solvent extraction has been the traditional method for the analysis of insect cuticular compounds [24,26,36,37,38]. The process is straightforward: the insect is placed into a nonpolar solvent, such as methylene chloride, ether, pentane, or hexane, during which time the biological compounds leach into the liquid. Those compounds may then be concentrated and analyzed by gas chromatography-mass spectrometry (GC-MS) or by high performance liquid chromatography (HPLC).

Previous work has been done demonstrating the discriminating power of chemical profiles for blow fly larvae and pupae [26,39,40,41,42]. Headspace solid phase microextraction (SPME) experiments of a single species (*Calliphora vicina*) utilized a polydimethylsiloxane/divinyl benzene/carboxen (PDMS/DVB/CAR) SPME fiber to collect hydrocarbons, fatty acids, and sterols from larvae, and a PDMS/CAR SPME fiber to do the same for pupae [39]. The presence and absence of the compounds were noted and the qualitative data was analyzed by agglomerative hierarchical clustering (AHC). The results in this case indicated that the chemical profiles of larvae and pupae were dissimilar, as were the profiles of pupae of differing ages [39].

The purpose of this project was to interrogate the chemical profiles that could be obtained from pupae by solvent extraction followed by total vaporization solid-phase microextraction (TV-SPME) [43]. The advantage of this method over headspace or immersion SPME is the elimination of the need to determine the optimal organic solvent, and that extracts do not need to be filtered reducing buildup and potential contamination of the GC column. Furthermore, TV-SPME can add additional selectivity and sensitivity based on fiber choice, and requires no changes in instrumentation.

Four species of blowfly were investigated: *Cochliomyia macellaria*, *Lucilia cuprina*, *Lucilia sericata*, and *Phormia regina*. The objectives were to: (i) determine whether the chemical profiles could be used to effectively discriminate between different species; (ii) to determine the extent to which genetic variation affected the chemical profile and the integrity of these determinations; and, (iii) to examine the effects of various abiotic variables on the chemicals extracted from the flies. These scenarios were used to produce a model of blow fly species identifications.

## 2. Materials and Methods

### 2.1. Rearing of Fly Colonies and Collection of Specimens

Colonies of *Phormia regina*, *Cochliomyia macellaria*, *Lucilia sericata*, and *Lucilia cuprina* were reared through ten successive generations (G). At the G1 and G10 generations, 19 × 100 maggots were transferred into a Dixie cup containing 50 g chicken liver, which were in turn placed inside 19 separate glass mason jars half-filled with a pupation substrate. The mason jars were kept in an incubator maintained at 25 °C temperature and 60% humidity. Seven to ten days were allotted for the maggots to develop through their larval stages. At 1 d following the observation of pupation, all of the pupae were harvested from three randomly-selected jars; these pupae were stored separately in a freezer at −80 °C and classified as Timepoint 1. Samples were collected each additional 24 h following for a total of six time points (18 jars). This data was not used to estimate age, but rather to determine the consistency of the chemical profiles over time to accurately identify the species. The maggots in the 19th jar served as a control, the time of adult emergence was recorded, and the number of adult flies was compared against the number of puparia and unhatched pupae to ensure proper development.

To gauge the chemical effects wrought by changes in temperature, humidity, pupation substrate, and diet, the same experimental design as above was carried out using pupae of G10+ *Phormia regina* (Table 1). During this set of experiments, pupae were likewise collected over six timepoints.

### 2.2. Instrumental Analysis

A 6890 gas chromatograph coupled to a 5975 mass spectrometer (Agilent, Santa Clara, CA, USA) served as the principal instrument, with autosampler functionality provided by an MPS2 (Gerstel, Mülheim an der Ruhr, Germany). The column was a J&W DB-5ms (30 m × 0.25 mm × 0.25 μm). All GC-MS analyses utilized H_2_ carrier gas with a flow rate of 2.5 mL/min operated in splitless mode, with a scan range of *m*/*z* 40–550.

For each timepoint, three haphazardly selected pupae from each of the three jars were thawed and extracted in 1 mL pentane. After 4 day, the extracted solution was siylated and submitted to GC-MS analysis via the TV-SPME method described [43]. This method provided excellent sensitivity an order of magnitude greater than what could be achieved by liquid injection, allowing for the analysis of liquid extracts without a reconstitution step.

### 2.3. Statistical Analysis

Following instrumental analysis, a qualitative assessment of the chromatograms was undertaken, retention times converted to retention indices (RI), and 26 compounds of interest were identified across all four species (see Results). Compounds were identified based upon their retention times and mass spectra. Authentic standards of the most common free fatty acids, squalene and cholesterol, were also analyzed alongside unknown samples. For compounds for which we did not have a standard, a computerized search of the National Institute of Standards and Technology (NIST) mass spectral database was conducted. In these cases, only forward match scores greater than 800/1000 were considered probative. The mass spectra for these compounds were compiled into an Automated Mass Spectral Deconvolution and Identification System (AMDIS) library, and automated peak integration proceeded using AMDIS mass spectral deconvolution software. The peak areas were normalized to the square root of the sum of squares for all compounds of interest in the chromatogram.

The normalized dataset was investigated by principal component analysis (PCA) and discriminant analysis (DA) to illuminate the underlying trends among the variables and timepoints. Therefore, a complex system of 26 variables (i.e., 26 compounds of interest) can be efficiently described by a system of three PCs, which can themselves be plotted on a Cartesian coordinate plane, thereby aiding in the visualization of the data.

DA is another technique whose purpose is to visualize groupings in the data, as well as to predict and ascribe group membership for new samples. DA strives to maximize the discrimination among different groups. The group memberships are pre-defined, but the integrity of these memberships can be assessed by a leave-one-out cross-validation, which is reported in a table known as a confusion matrix.

## 3. Results and Discussion

### 3.1. Determination of Species

The relative abundance of the 26 compounds of interest for the G1 generations are presented in a heat map in Table 2. Wherever possible, the identity of the compound has been reported. However, due to similar retention times and indistinguishable mass spectra, the exact identities of some of the hydrocarbons remain elusive. These have been compared against the retention times of an n-alkane mix and differentiated based upon their retention indices. Similarly, the compounds labeled “14:1 FFA” and “18:1 FFA” are mono-unsaturated 14:1 and 18:1 free fatty acids, but the precise location of the double bond is unknown.

There is a high degree of qualitative similarity between the four species, particularly with respect to the expression of fatty acids. For example, palmitoleic acid, palmitic acid, linoleic acid, and oleic acid are observed at relatively high levels in all samples for all species. Substantially more variability is evident in the hydrocarbon compound class, with certain alkanes commonly seen in some species, yet wholly absent in other species. For example:

1. alkane A (retention index (RI) = 1465) was only present in *L. sericata*.
2. pentadecane, alkane C (RI = 2800), alkane F (RI = 2934), and alkane G (RI = 3084) were present in all species except *P. regina*.
3. alkane D (RI = 2867) was only present in *C. macellaria.*

Less obvious in Table 2, but still just as important to the statistical analysis, are the more nuanced quantitative differences in the detection of these compounds among the different species (i.e., differences in the relative amounts of each of the major compounds). It is often customary to reduce the dimensionality of a dataset first via PCA prior to attempting DA. This is typically done to sidestep one of the inherent limitations of DA, where each grouping must possess more observations (i.e., pupa exemplars) than variables (i.e., compounds of interest, or principal components thereof). A preliminary round of PCA therefore serves as a convenient way to reduce the number of variables whilst still capturing a sizeable amount of the variance in the data. For this data set, PCA of the G1 generation (data not shown) tended to over-emphasize the differences between *Lucilia cuprina* and *Lucilia sericata* at the expense of the differences between *Cochliomyia macellaria* and *Phormia regina*, and therefore obfuscated more than it aided.

**Figure 1:**
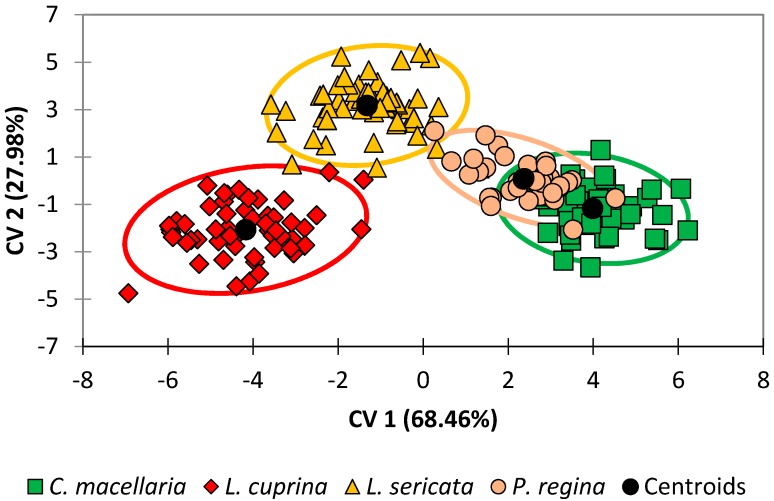
Discriminant analysis of the original variables for all four species, G1 generation, at all timepoints. The ellipses correspond to the 95% confidence limits of each species based upon its centroid and distribution of observations in the canonical variate (CV) space.

Improved discrimination of species was achieved in this study by performing DA on the dataset directly without the intermediate PCA step. DA results for the G1 generation were prepared by this method and an observations plot is shown in Figure 1. This plot illustrates the two species that are readily differentiable (*L. cuprina* and *L. sericata*) and two species that are more apt to be confused (*C. macellaria* and *P. regina*).

**Figure 2:**
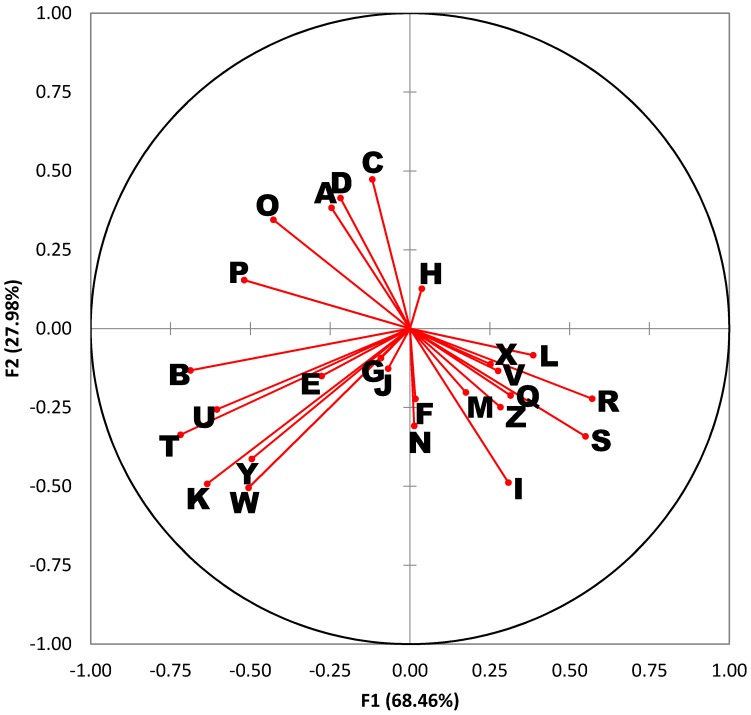
Variables plot for discriminant analysis (DA) results in Figure 1. Identities for the rays projected from the origin correspond to the lettered compounds in Table 2.

A variables plot is also provided in Figure 2. This type of plot serves as a “roadmap” for interpreting the DA results in Figure 1. Each ray in the variables plot represents one of the 26 compounds of interest. Rays that form an acute angle represent compounds whose abundances are highly correlated. For example, rays I, R, and S, correspond to myristic acid, octadecenoic acid (18:1), and arachidonic acid, which tend to rise and fall together. Similarly, rays T, U, W, and Y, represent long-chain alkanes (waxes) that are also correlated. Rays that form obtuse angles are highly anti-correlated. For example, linoleic acid (ray O) and arachidonic acid (ray S) are negatively correlated. Rays at right angles reflect compounds with little or no correlation.

The quadrants of the variables plot (Figure 2) are also directly comparable to the quadrants of the corresponding DA chart (Figure 1). The length of each ray indicates the magnitude of the association. Long rays indicate variables that are important in describing the variation in sample profiles, whereas short rays that fall close to the origin are compounds that describe little to none of the variation. The distribution of the rays in Figure 2 is quite instructive. For example, rays B, K, T, U, W, and Y project into the lower left quadrant and tend to be found at higher levels in *L. cuprina*. Rays I, R, and S project into the lower right quadrant and tend to be found at higher levels in *P. regina* and *C. macellaria*. Lastly, rays A, C, D, O, and P project into the top left quadrant and tend to be found at higher levels in *L. sericata.*

Thus, the following observations are manifest:

- *Lucilia cuprina* is distinguishable by high concentrations of palmitoleic acid and several long-chain alkanes (alkane B, alkane C, alkane E, and alkane G).
- *Phormia regina* and *Cochliomyia macellaria* are distinguishable by high concentrations of myristic acid, 18:1 FFA, and arachidonic acid.
- *Lucilia sericata* is distinguishable by high concentrations of several alkanes, linoleic acid, and oleic acid.

Lastly, when the DA model for the G1 generation was tested using leave-one-out cross-validation (Table 3), classification accuracies ranged from 89% (*P. regina*) to 96% (*L. sericata*) with an overall classification accuracy of 93%. Whereas Figure 1 allows for a qualitative assessment of the discrimination of the four species, classification accuracy is a quantitative way to assess the level of confusion between classes. Therefore, it is worth noting that of the four species, *P. regina* and *C. macellaria* had the lowest accuracies. For *P. regina*, 75% (three out of four) of the misclassifications were due to *P. regina* observations erroneously classified as belonging to the *C. macellaria* species. Similarly, 75% (three out of four) of the misclassifications were due to *C. macellaria* observations erroneously classified as belonging to the *P. regina* species. Achieving an overall classification accuracy of greater than 90% is an excellent result and this is sound evidence for the theory that taxonomic information can be gleaned from the lipid profiles.

### 3.2. Effect of Genetic Diversity

A key issue this project sought to address was the effect of genetic diversity on a specimen’s chemical profile. Specifically, do G1 pupae yield a markedly different suite of compounds than pupae belonging to the G10 generation, whose genetic diversity has been homogenized over successive generations of inbreeding?

**Figure 3:**
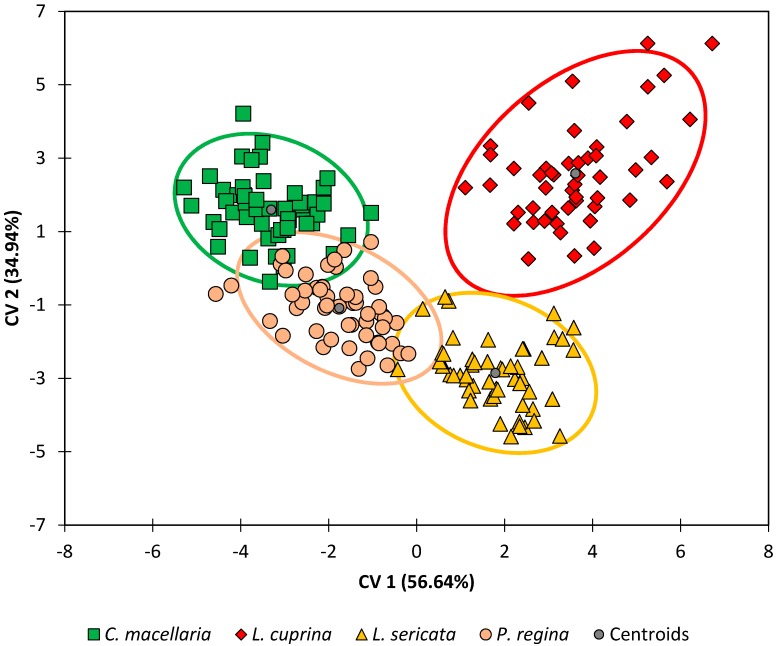
Discriminant analysis of all four species, G10 generation, at all timepoints.

**Figure 4:**
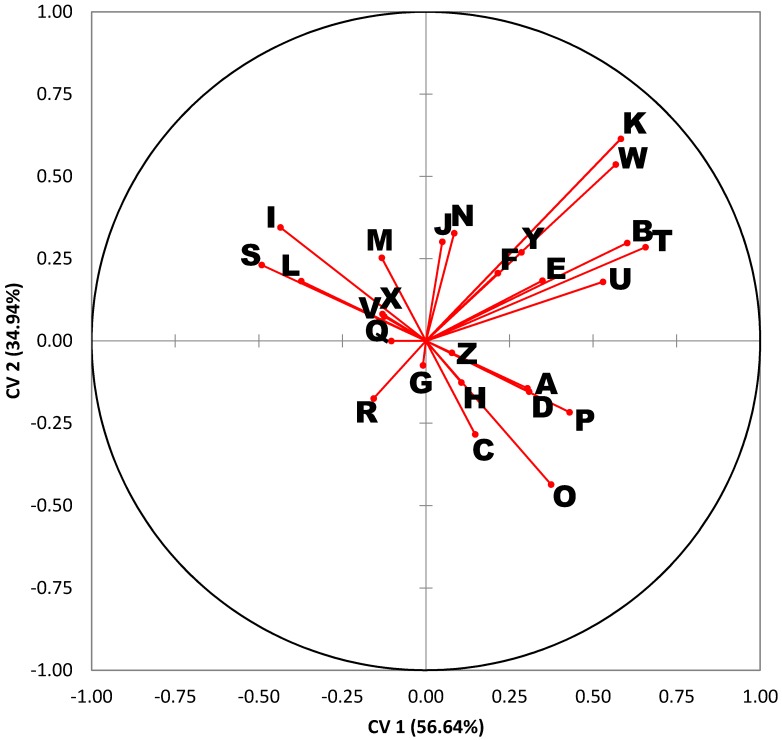
Variables plot for the discriminant analysis results in Figure 3. Identities for the projected rays from the origin correspond to the lettered compounds in Table 2.

When the results from the G10 generations were analyzed by DA (see Figure 3 and Figure 4 and Table 4), the results were like those for the G1 generation. Note that the orientation of the species within the DA plot is rotated ~180° relative to Figure 2. This is not as significant as the relative positions of the classes and the overall separation between species, which is more evident in this data set (Figure 3). The correlations between variables and the observations are also very similar (Figure 4), although rotated relative to Figure 2 as mentioned above. One exception was compound R (18:1 or octadecenoic acid), which was no longer strongly correlated with any of the other compounds.

Lastly, when the DA model for the G10 generation was tested using leave-one-out cross-validation (Table 4), classification accuracies ranged from 94% (*P. regina*) to 98% (*L. cuprina*) with an overall classification accuracy of 96%. In similar fashion to the G1 generation, most of the misclassifications for *P. regina*, 67% (four out of seven) were due to *P. regina* observations that were erroneously classified as belonging to the *C. macellaria* species, and vice versa. Similarly, all misclassifications for *C. macellaria* (two out of two) were due to *C. macellaria* observations that were erroneously classified as belonging to the *P. regina* species. The overall performance of the DA model is better for the G10 generation (96%) than it was for the G1 generation (93%). This small increase in accuracy may be explained by reducing the intra-species variation through repeated inbreeding.

DA was also used to attempt to discriminate samples hailing from the G1 and G10 generation of each species (i.e., *C. macellaria* G1 samples were compared to *C. macellaria* G10 samples, *L. cuprina* G1 samples were compared to *L. cuprina* G10 samples, etc.). When DA is applied to only two classes (as in a series of pair-wise comparisons), a Receiver Operating Characteristic (ROC) curve can be constructed. ROC curves depict the binary classification accuracy of the system, and the area under a ROC curve varies from 0.5 (no discrimination) to 1.0 (perfect discrimination).

The classification accuracy and ROC results for the G1 and G10 generations of all four species are shown in Table 5. The species whose G1 and G10 generations were the most distinguishable was *P. regina*, with a classification accuracy of 82.2% and a ROC area under the curve (AUC) of 0.88. Therefore, the effects of genetic homogenization were the most significant for this species. The species whose G1 and G10 generations were the least distinguishable was *L. sericata*, with a classification accuracy of 58.9% and ROC AUC of 0.65. Therefore, the effects of genetic homogenization were the least significant for this species.

DA was also performed by treating samples of G1 pupae as unknowns and classifying them per the DA model constructed from G10 pupae. This models a situation where a laboratory would have a database of lipid profiles taken from flies that could reproduce and inbreed for 10 generations. That same laboratory would then take case samples, analyze them immediately, and classify them per this database. If genetic differences are irrelevant, then the G1 “casework” pupae would be expected to be grouped per their species with near 100% accuracy, as was seen for the G10 “database” generation provided in Table 4. Poor classification accuracy, conversely, would indicate that the model is indeed betrayed by genetic differences. The results are shown in Table 6.

Although the confusion matrix shows lower classification accuracies across the board (when compared to Table 4), the accuracies for *L. cuprina* and *L. sericata* are excellent (>90%) and the accuracy for *C. macellaria* is very good (89%), despite the mismatch between the training set (G10 samples) and the test set (G1 samples). It is also interesting to see that the species whose G1 and G10 generations were reliably differentiated in Table 5 (i.e., *P. regina*), and therefore, exhibited the most significant chemical changes, had low classification accuracies (75%) in this example. Conversely, a species whose G1 and G10 generations were not differentiated in Table 5 (e.g., *L. sericata*), and therefore, exhibited the least significant chemical changes, had high classification accuracy (93%). It should be noted that no *L. sericata* were classified as *L. cuprina*, and 2 of 54 (7%) of *L. cuprina* were classified as *L. sericata*. This is important as both species belong to the same genus and share a close evolutionary history.

Based on these results, it appears that G10 pupae may be a suitable model for G1 pupae, if the effect of genetic homogenization is not significant. For species such as *P. regina*, genetic homogenization does indeed alter the chemical profile in a statistically-significant way. Future researchers seeking to extrapolate useful information about G1 and wild-type populations based on data gained from advanced generations are cautioned to bear this in mind.

### 3.3. Effects of Abiotic Variables (P. regina)

DA was carried out and confusion matrices were generated for the data from the studies of temperature, humidity, substrate, and diet. Although overall classification accuracies of 60% or more were achieved for all four experiments, in most cases the actual differences observed among the classes were marginal. For example, almost no difference in the lipid profile was manifest among specimens allowed to pupate in sawdust, in sand, or in vermiculite (data not shown). Classification accuracies were therefore poor, with a third of samples misclassified in the cases of pupae reared in sawdust and sand, and half of all samples in the case of vermiculite. In these cases, only the data in the experiment (for example, the pupation substrate) were used, therefore, it appeared that it did not matter what the pupation substrate was, there were no distinct classes formed.

Results for pupae reared under different humidity conditions were almost as poor (data not shown), with discrimination only achieved for the sample set reared under normal 55% humidity conditions. This is perhaps a surprising result, as the cuticular hydrocarbons of insects are known to play a part in resistance to desiccation, and might be expected to vary in response to adverse environmental conditions such as low humidity. It should be noted that although discrimination could not be achieved in these cases, this is, in fact, beneficial. In the context of insect specimens recovered from a crime scene, it is to the advantage of an investigator if the chemical profiles do not vary significantly with temperature, humidity, and other uncontrollable factors. A reliable method for age and species determination that is not undermined or biased by biotic and abiotic variables is the gold standard for any analyst. Therefore, the lack of discrimination is, in this case, the desired result.

Temperature appeared to have more of an impact on the lipid profiles, although its influence was still relatively small, as attested by the poor classification accuracies of 41.7% and 62.3% for pupae reared at 15 °C and 20 °C, respectively (data not shown). More substantial chemical differences were observed at 25 °C and 30 °C. In general, lauric acid, myristic acid, pentadecanoic acid, palmitic acid, and alkane G increased with temperature.

**Figure 5:**
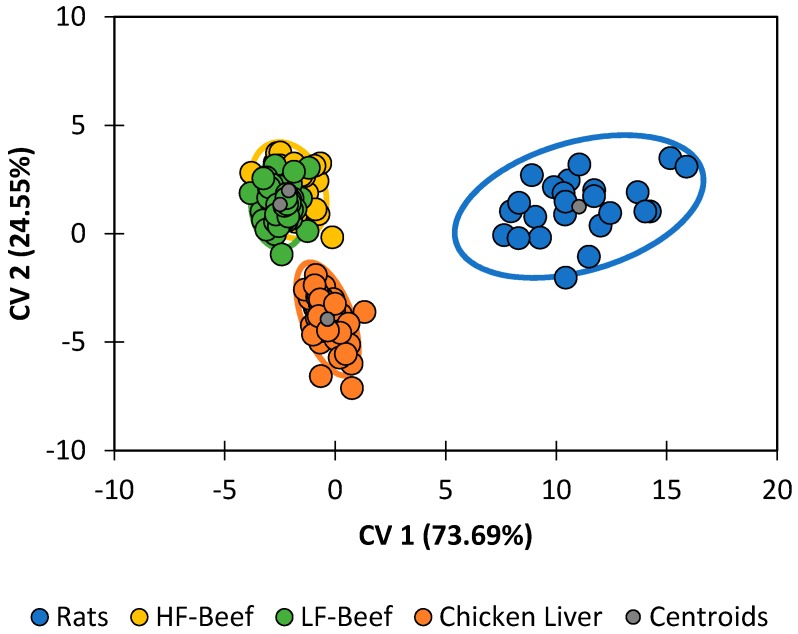
Discriminant analysis of *P. regina* pupae reared on various diets.

Diet was the single-largest contributing factor to differences in the lipid profile (Figure 5). Classification accuracies (Table 7) were 100% for pupae reared on chicken liver and 87.5% for pupae reared on feeder rats. The statistical model was unsuccessful at distinguishing between pupae raised on high-fat ground beef and pupae fed on low-fat ground beef. Neglecting that minor distinction, pupae raised on ground beef were still correctly categorized 100.00% of the time. The following trends are clear:

- Pupae reared on chicken liver have lipid profiles with higher quantities of palimitic acid, palimitoleic acid, unknown alkane B, and cholesterol.
- Pupae reared on ground beef have lipid profiles with higher quantities of lauric acid, myristic acid, palmitelaidic acid, linolenic acid, and stearic acid.
- All other compounds appear in greatest abundance in feeder rats.

The most meaningful takeaway from this series of experiments is that, which the exception of diet, changes in any given abiotic factors did result in reliably differentiable classes for *P. regina*. However, each factor had a larger capacity to obfuscate accurate species determinations when comparing to a population of *P. regina* reared under normal conditions. When each chemical profile under every single differing abiotic condition was validated using discriminant analysis with the four species (G10) as the training set, most species classifications were less than 50% correct (Table 8). However, when comparing the control conditions (these conditions are identical to the G10 conditions) with expected classification accuracies of >82% (see Table 5), only 37.7% classification accuracy was recovered. This is indicative that conditions changed between the G10 rearing conditions and that of the abiotic factor experiments conducted sometime later. Nevertheless, it should be noted that classification accuracy using discriminant analysis is only as good as the similarity between the training and validation data sets. In this case, the data sets differ, thus obscuring an accurate species identification. Our data indicate that diet has the greatest impact (with classification accuracies less than 2%), whereas temperature and pupation substrate appear to be most like the control profiles (Table 8). This is not surprising; given that pupae produce lipids in their cuticles for the protection against desiccation and predation, temperature and pupation substrate (our substrates were all dry) should not have much of an effect. However, humidity (different lipids/lipid ratios would be needed to protect the pupa), and diet (the lipids would be obtained in different ratios) would significantly influence the profiles.

## 4. Conclusions

This study aimed to better understand how biotic (reduced genetic variation) and abiotic factors (temperature, humidity, pupation substrate, and diet) influence the uses of chemical analyses for species identification of forensically important pupae. Considerable chemical differences were documented among *Cochliomyia macellaria*, *Lucilia cuprina*, *Lucilia sericata*, and *Phormia regina* as a function of species and genetics. Classification accuracies of 89–94% were obtained for the G1 generation, and classification accuracies of 93%–98% were obtained for the G10 generation. The compounds most correlated and anti-correlated with species were identified. Data from the G10 generations were shown to be a suitable model for G1 pupae, although performance on *P. regina* suffered, with classification accuracy falling to 75%. However, genetic homogenization was found to have an impact on the observed chemical profiles, which should serve as a cautionary note for future researchers hoping to extract data with real world relevance from colonies at advanced generations. Temperature and pupation substrate were found to have minimal impact on the lipid profiles, whereas small chemical changes were observed with changes in humidity, and significant changes were observed with variations in diet.

If comparing the classification accuracies of the different abiotic conditions relative to the control, it is immediately clear that diet plays the most important role in the composition of lipid profiles in developing pupae (Table 8). Therefore, in “real-world” scenarios where pupae are recovered from a scene but their diet is unknown, we suspect that using chemical profiles to determine species is not ideal, and thus more research is necessary. Although we used a single classification method here (discriminant analysis), other methods such as (Soft Independent Modeling of Class Analogy (SIMCA), Neural networks or Bayesian classifiers, etc.) could change the classification accuracies presented here. However, the effect of diet is a biochemical process and not one that could be easily corrected for via statistical methods. It is important that additional data be collected to establish a greater understanding of the changes in chemical profiles over time under different conditions to establish a reliable database for species identification purposes.

## Figures and Tables

**Table 1:** Experimental design for the evaluation of abiotic factors of temperature, humidity, feeding, and pupation substrates on lipid profiles for the determination of age of *Phormia regina* pupae. L:D: Light: Dark; CL: chicken liver; LFB: low fat beef; HDB: high fat beef; SD: sawdust; Ver: vermiculite.

Abiotic Factor	Temperature (°C)	Humidity (%)	Feeding Substrate	Pupation Substrate
Temperature	15	55	CL	SD
	20	55	CL	SD
	25	55	CL	SD
	30	55	CL	SD
Humidity	25	40	CL	SD
	25	70	CL	SD
	25	85	CL	SD
Larval Feeding Substrate	25	55	LFB	SD
	25	55	HFB	SD
	25	55	Rat	SD
Pupation Substrate	25	55	CL	SD
	25	55	CL	Sand
	25	55	CL	Ver

**Table 2:** Heat map depicting the log of the normalized peak areas for the G1 generation of *C. macellaria* (*n* = 53), *L. cuprina* (*n* = 54), *L. sericata* (*n* = 54), and *P. regina* (*n* = 36) across all time points. Twenty-six compounds were selected for statistical analysis. The color scale is logarithmic, varying from black (10^−7^) to red (10^0^). “TCAIE” (Compound E) is 2,2,4-trimethyl-3-carboxyisopropyl- pentanoic acid isobutyl ester. FFA: free fatty acid; ND: not determined.

Identifier	Compound	*C. macellaria*	*L. cuprina*	*L. sericata*	*P. regina*	Identifier	Compound	*C. macellaria*	*L. cuprina*	*L. sericata*	*P. regina*
A	Tetradecane	−3.80	−2.79	−2.32	−5.12	N	Linolenic Acid	−1.82	−1.84	−2.16	−2.09
B	FFA	ND	−2.73	−3.05	ND	O	Linoleic Acid	−0.60	−0.47	−0.42	−0.53
C	Alkane A	ND	ND	−2.94	ND	P	Oleic Acid	−0.32	−0.24	−0.24	−0.33
D	Pentadecane (C_15_H_32_)	−4.94	−2.74	−2.13	ND	Q	Stearic Acid	−1.83	−2.01	−2.18	−1.71
E	TCAIE	−2.07	−1.79	−2.04	−2.88	R	18:1 FFA	−2.43	−3.31	−3.44	−2.50
F	Lauric Acid	−2.32	−2.34	−2.48	−2.48	S	Arachidonic Acid	−1.23	−1.51	−1.62	−1.32
G	Isopropyl Myristate	−3.14	−2.72	−3.31	−3.46	T	Alkane B	−3.31	−1.83	−2.36	−2.65
H	14:1 FFA	−3.21	−3.31	−3.14	−3.24	U	Alkane C	−5.86	−3.45	−3.99	ND
I	Myristic Acid	−1.16	−1.28	−1.61	−1.22	V	Alkane D	−3.24	ND	ND	ND
J	Pentadecanoic Acid	−2.58	−2.30	−3.13	−2.97	W	Alkane E	−2.31	−1.57	−2.85	−3.23
K	Palmitelaidic Acid	−1.51	−0.99	−1.47	−1.42	X	Alkane F	−3.26	−5.26	−4.63	ND
L	Palmitoleic Acid	−0.27	−0.35	−0.34	−0.25	Y	Alkane G	−5.02	−3.00	−6.27	ND
M	Palmitic Acid	−0.22	−0.25	−0.29	−0.24	Z	Cholesterol	−2.56	−2.98	−3.52	−2.88

**Table 3:** Leave-one-out confusion matrix for the G1 generation chemical profiles.

From/To	*C. macellaria*	*L. cuprina*	*L. sericata*	*P. regina*	Total	% Correct
*C. macellaria*	49	2	0	3	53	92.5
*L. cuprina*	0	62	3	0	54	94.4
*L. sericata*	0	3	52	0	54	96.3
*P. regina*	3	0	1	32	36	88.9

**Table 4:** Leave-one-out confusion matrix for the G10 generation chemical profiles.

From/To	*C. macellaria*	*L. cuprina*	*L. sericata*	*P. regina*	Total	% Correct
*C. macellaria*	52	0	0	2	54	96.3
*L. cuprina*	0	48	1	0	49	98.0
*L. sericata*	0	0	52	2	54	96.3
*P. regina*	2	0	1	51	54	94.4

**Table 5:** Classification accuracy and area under the curve (AUC) for the G1 and G10 pupae of each species.

G10 Generation					
	From/to	*C. macellaria*	*L. cuprina*	*L. sericata*	*P. regina*
G1 Generation	*C. macellaria*	68.9% (0.832)			
	*L. cuprina*		73.5% (0.839)		
	*L. sericata*			58.9% (0.648)	
	*P. regina*				82.2% (0.883)

**Table 6:** Leave-one-out confusion matrix for the G1 generation chemical profiles.

G10 Generation						Total	% Correct
	From/To	*C. macellaria*	*L. cuprina*	*L. sericata*	*P. regina*		
G1 Generation	*C. macellaria*	47	0	0	6	53	88.7
	*L. cuprina*	2	49	2	1	54	90.7
	*L. sericata*	0	0	52	2	54	93.0
	*P. regina*	7	0	2	27	36	75.0

**Table 7:** Leave-one-out confusion matrix for *P. regina* pupae reared on larval diets.

From/To	Rats	HF-Beef	LF-Beef	Chicken Liver	Total	% Correct
Rats	21	2	0	1	24	87.5
HF-Beef	0	36	17	0	53	67.9
LF-Beef	0	13	41	0	54	75.9
Chicken Liver	0	0	0	53	53	100

**Table 8:** Leave-one-out confusion matrix for *P. regina* larvae reared under different abiotic conditions and to which species they assign. The control abiotic conditions were 25 °C, 55% r.h., chicken liver and sawdust—the same conditions as the G10 generation. Only the abiotic condition in column one changed, other conditions remained as in the control condition.

From/To	*C. macellaria*	*L. cuprina*	*L. sericata*	*P. regina*	Total	% Correct
Rats	0	3	21	0	24	0
HF-Beef	2	50	0	1	53	1.9
LF-Beef	2	51	0	1	54	1.9
15 °C	0	8	10	6	24	25.0
20 °C	4	8	18	23	53	43.4
30 °C	20	5	12	16	53	30.2
40% r.h.	0	9	21	12	42	28.6
70% r.h.	3	26	19	4	52	7.7
85% r.h.	0	15	25	13	53	24.5
Sand	2	8	18	26	54	48.1
Vermiculite	0	10	11	23	44	52.3
CONTROL	0	6	27	20	53	37.7
